# Assortative breeding experiment in a songbird suggests telomere length is determined during early life rather than at conception

**DOI:** 10.1038/s41598-025-23517-7

**Published:** 2025-10-21

**Authors:** Ye Xiong, Julian Melgar, Michael Tobler, Dennis Hasselquist

**Affiliations:** 1 Department of Biology, Lund University, Ecology Building, Lund, SE-223 62 Sweden; 2 Department of Ecology and Evolution, University of Lausanne, Biophore, Lausanne, CH-1015 Switzerland

**Keywords:** Fetal programming, Zebra finch, Early development, Sex differences, Ageing, Ecological genetics, Ecology, Evolutionary ecology

## Abstract

Telomere length (TL) early in life often shows high heritability and may predict telomere shortening later in life and life expectancy. Yet, there is limited data about what influences TL and TL change at early developmental stages. It is debated whether early-life TL is determined at conception or shaped by early environmental conditions. Here, we investigate whether TL and telomere shortening are set close to conception. We assortatively paired zebra finches (*Taeniopygia guttata*) based on their TL when nestlings, forming two parental pair groups with either ‘short’ or ‘long’ TL. We then measured TL in the offspring of these pairs at the embryo and nestling stages. In embryos, TL did not differ between offspring from the two parental pair groups. However, in nestlings, particularly in sons, offspring TL matched parental TL. Our results suggest that early-life TL itself is not determined at conception. Instead, telomere shortening rate before and just after hatching appears to determine early postnatal TL. These findings highlight that early development is critical for telomere shortening during early life stages and that it may be a key process underlying the similarity in early-life TL between parents and offspring, potentially affecting telomere dynamics throughout life.

**Supplementary Information:**

The online version contains supplementary material available at 10.1038/s41598-025-23517-7.

## Introduction

Telomeres are repeated sequences of DNA at the end of linear chromosomes^1^. They preserve genome stability by acting as a protective structure for coding DNA thus preventing chromosome degradation (e.g., due to oxidative stress) and chromosome fusions^2^. Telomeres also typically shorten at each cell division and, thus, with age. Moreover, increased telomere shortening and severely shortened telomere length (TL) are associated with accelerated senescence and disease susceptibility later in life^3–5^. There is evidence that an individual’s telomere length early in life (prenatal or early postnatal) is correlated with telomere shortening later in life, i.e., that individual differences in early life TL carry over into adulthood^6–9^. Moreover, it has been found that TL early in life can predict lifespan and be positively correlated with proxies of fitness^10–13^. In line with these findings, it has been proposed that early TL could predetermine life trajectories and life history decisions^14–16^.

However, there is currently still a lack of data on TL and telomere shortening regarding the very early developmental stages^17^, and we therefore know relatively little about which factors that affect/predict TL and TL change at the early life stages. Consequently, there is debate about the role of early life environment versus genetic inheritance of TL^18^. Some studies show that early life environmental conditions (e.g., developmental stress, temperature effects, nutrient availability, pollutants) can affect TL and telomere shortening^6,7,19–25^, and it has been suggested that this may have downstream effects on lifespan and performance later in life^14–16^. In contrast, other studies show that genetic inheritance of TL in some species is considerable^11,18,26,27^, supporting the idea that individual TL is largely determined at conception. In this study, we aimed to test whether individual TL and telomere dynamics are set at a very early stage in life (i.e., close to conception).

Since measurement of prenatal TL typically requires invasive procedures that may harm the embryo, it is very difficult to measure both pre- and postnatal TL in the same individual. This complicates investigation of whether individual telomere trajectories are set up already during very early stages of embryonic development. However, cross-sectional data (with measurements of pre- and postnatal TL in offspring of the same species) can also provide valuable insights. If prenatal TL patterns are carried over into postnatal life stages, this could indicate that TL trajectories are set up already from early development onwards. In birds, collection of embryos and measurement of their TL is feasible as they develop outside the mother. However, we are aware of only two studies that have measured pre- and postnatal TL in birds^6,7^. Stier et al.^7^ found that different incubation regimes for Japanese quail (*Coturnix japonica*) eggs had no effect on embryo TL, but resulted in TL differences at the chick stage and into adulthood. This supports the idea that early life environmental conditions play an important role for telomere dynamics. Unfortunately, the parents’ TL was unknown in this study and therefore any genetic contribution to prenatal and early postnatal TL could not be assessed. Noguera et al.^6^ investigated TL in embryos and nestlings of zebra finches (*Taeniopygia guttata*). They found that TL in 3-day-old embryos markedly decreased with within-clutch ovulation order and that this ovulation order effect was still evident in adulthood. Thus, this suggests a strong genetic or non-genetic parental effect on TL during early embryonic development. In addition, previous studies on zebra finches have found that TL in this species seems to be highly heritable^27^ and strongly affected by parental and grand-parental age^28,29^. This could imply that TL and subsequent telomere dynamics (most often net shortening) are determined already at conception.

**Fig. 1:**
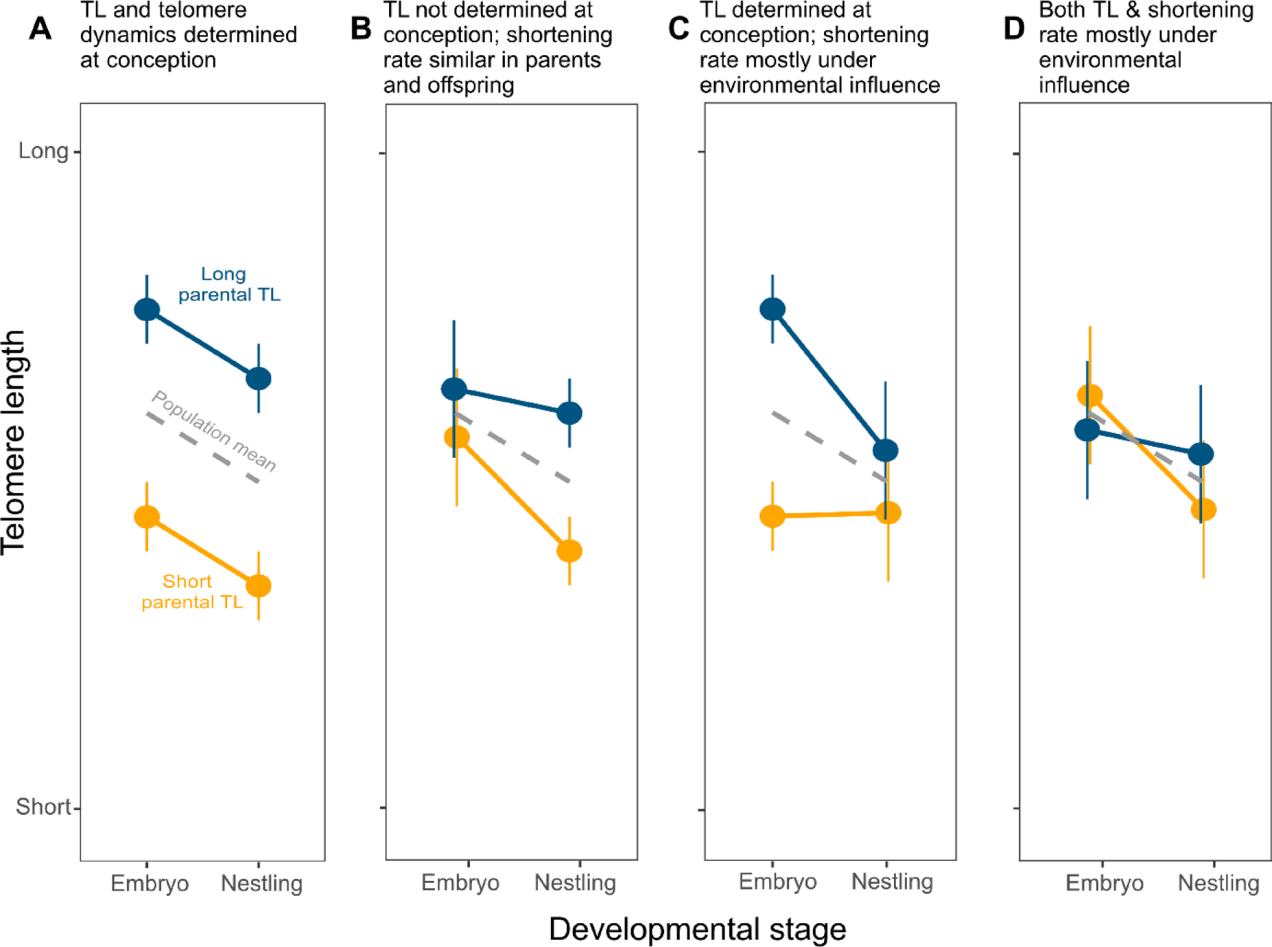
Schematic figures of alternative outcomes that can be predicted based on the experimental set-up. Parental pairs were mated assortatively based on the telomere length they had as nestlings (10 days post hatch; nTL); both the father and the mother of a brood had either short or long nTL (see methods for details). (**A**) *Parallel patterns*: TL and telomere dynamics are determined at conception. Offspring TL matches parental pair group (i.e., long or short nTL) both at the embryo and the nestling stage; (**B**) *Diverging patterns*: TL itself is not determined at conception, which means that TL of embryos does not reflect parental pair group. However, the offspring TL at the nestling stage should match that of their parental pair group if the mechanisms that control telomere shortening during the pre- and early postnatal periods operate in the same way in parents and offspring. (**C**) *Converging patterns*: TL is determined at conception, but large environmental effects have a main effect on TL shortening rate resulting in no match in TL between parents and offspring at the nestling stage; (**D**) *Stochastic patterns*: Offspring TL does not match the parental group at any of the developmental stages due to strong environmental effects acting both at the pre- and postnatal stages.

Here, we test the prediction that TL and telomere dynamics are determined at conception by applying a novel (assortative) breeding design to examine whether embryo TL and nestling (i.e., early postnatal) TL mirror parental early postnatal TL when keeping environmental effects constant. Rather than manipulating environmental variables that might affect TL during early development^6,21^, we manipulated the genetic and/or the non-genetic parental components that may affect offspring TL by creating pair constellations that were based on parental TL measured at the nestling stage. We assortatively paired adult zebra finches based on the TL they had as nestlings (measurements taken at day 10 post-hatch, hereafter abbreviated nTL), creating two **parental pair groups** that consisted of birds that had either **short** or **long** nTL. We predicted that if telomere trajectories are set already during embryonic development (i.e., close to conception) and environmental effects during early development have no or only minor impact, both embryo and nestling TL would match the TL of their parental pair group (parallel patterns, Fig. 1A). We also envisioned an alternative outcome in which postnatal differences in TL are more strongly influenced by (non-genetic) parental effects during the embryonic period (i.e., TL and TL trajectories are determined during early development but not at conception). In this case, we predicted a weak or no match between offspring embryo TL and parental pair group (long vs. short nTL), but a good match between parental pair group and the TL of their offspring at the nestling stage (diverging patterns, Fig. 1B). Given the high heritability of (early life) TL in zebra finches, we considered parallel and diverging patterns the two most likely outcomes. However, two additional scenarios may be possible (although in our view less likely). In a third scenario (converging patterns, Fig. 1C), offspring TL matches parental nTL at the embryo stage but not at the nestling stage (e.g., due to a heritable component of TL at conception but strong influence of environmental effects on the rate of telomere change would disconnect offspring and parental TL at the postnatal stage), and in a fourth scenario (stochastic patterns, Fig. 1D), offspring TL does not match the parental nTL group at either the embryo or the nestling stages, suggesting that strong stochastic environmental effects are acting at both the pre- and postnatal stages.

Because previous studies of birds, including zebra finches, have found that both parental age^30^ and offspring sex^31^ can predict early-life TL, we also examined whether embryo and nestling TL in our dataset (which included two different age cohorts of parental pairs) was affected by these factors, and whether effects might differ with respect to parental pair group.

## Results

**Fig. 2:**
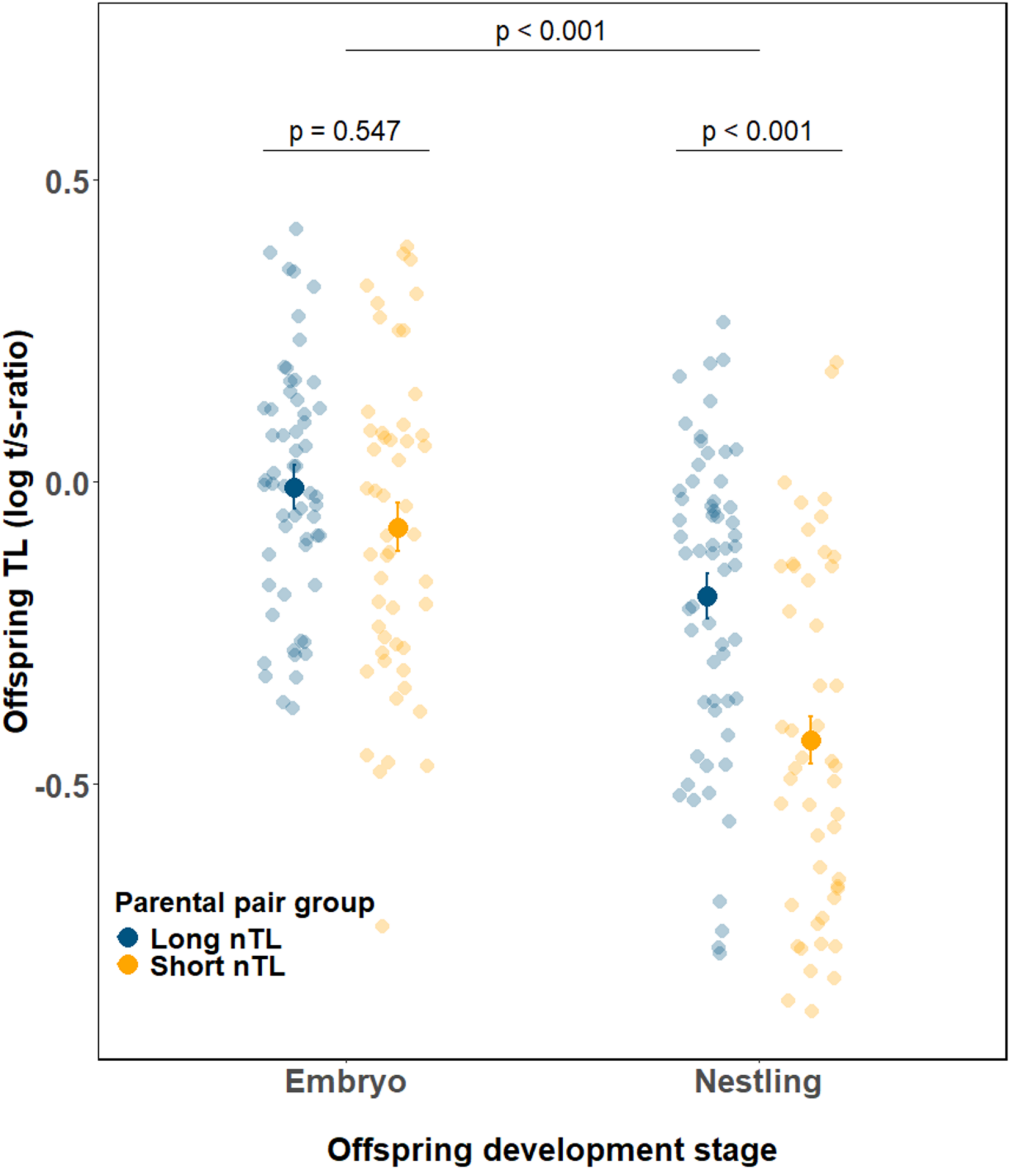
Offspring telomere length at different developmental stages (embryo vs. nestling) in relation to parental pair group (‘short’ parental pair group = parents with **short** nTL (orange, embryo *n* = 51, nestling *n* = 48); ‘long’ parental pair group = parents with **long** nTL (blue, embryo *n* = 59, nestling *n* = 60)). Large points and error bars represent mean ± S.E. of log-transformed t/s-ratio values, while semi-transparent small points show individual raw data. Significant differences between offspring developmental stages, and between parental pair groups in nestlings were determined using *post hoc* pairwise comparisons of least square means. nTL = the parents’ TL when they were nestlings.

Our assortative mating treatment significantly influenced offspring TL through an interaction between parental pair group and developmental stage (F_1,201.7_ = 6.51, *p* = 0.011 Fig. 2, Supporting information Table S1). On average, embryos had 30.5% longer telomeres than nestlings (based on the back-transformed LS-means differences (log-scale LS-means ± SE for embryos vs. nestlings: -0.042 ± 0.027 vs. -0.308 ± 0.027; estimate ± SE = 0.266 ± 0.034, t_206_ = 7.93, p < 0.001. Fig. 2). When we conducted separate analyses for the embryo and the nestling stages, we found that parental pair group had an effect on the TL of nestlings, but not on the TL of embryos. At the embryo stage, TL of offspring from parents with long nTL did not differ statistically from TL of offspring from parents with short nTL (LS-means ± SE: -0.017 ± 0.041, and − 0.054 ± 0.044, respectivel;; F_1,31.7_ = 0.37, *p* = 0.546; Fig. 2, Supporting information Table S2). At the nestling stage, offspring TL matched their parental pair group, with nestlings from parents with long nTL having longer telomeres than those from parents with short nTL (LS-means ± SE: -0.189 ± 0.045 vs. − 0.434 ± 0.048; estimate ± SE = 0.245 ± 0.066, t_35_ = 3.70, p < 0.001 ; Fig. 2).

**Fig. 3:**
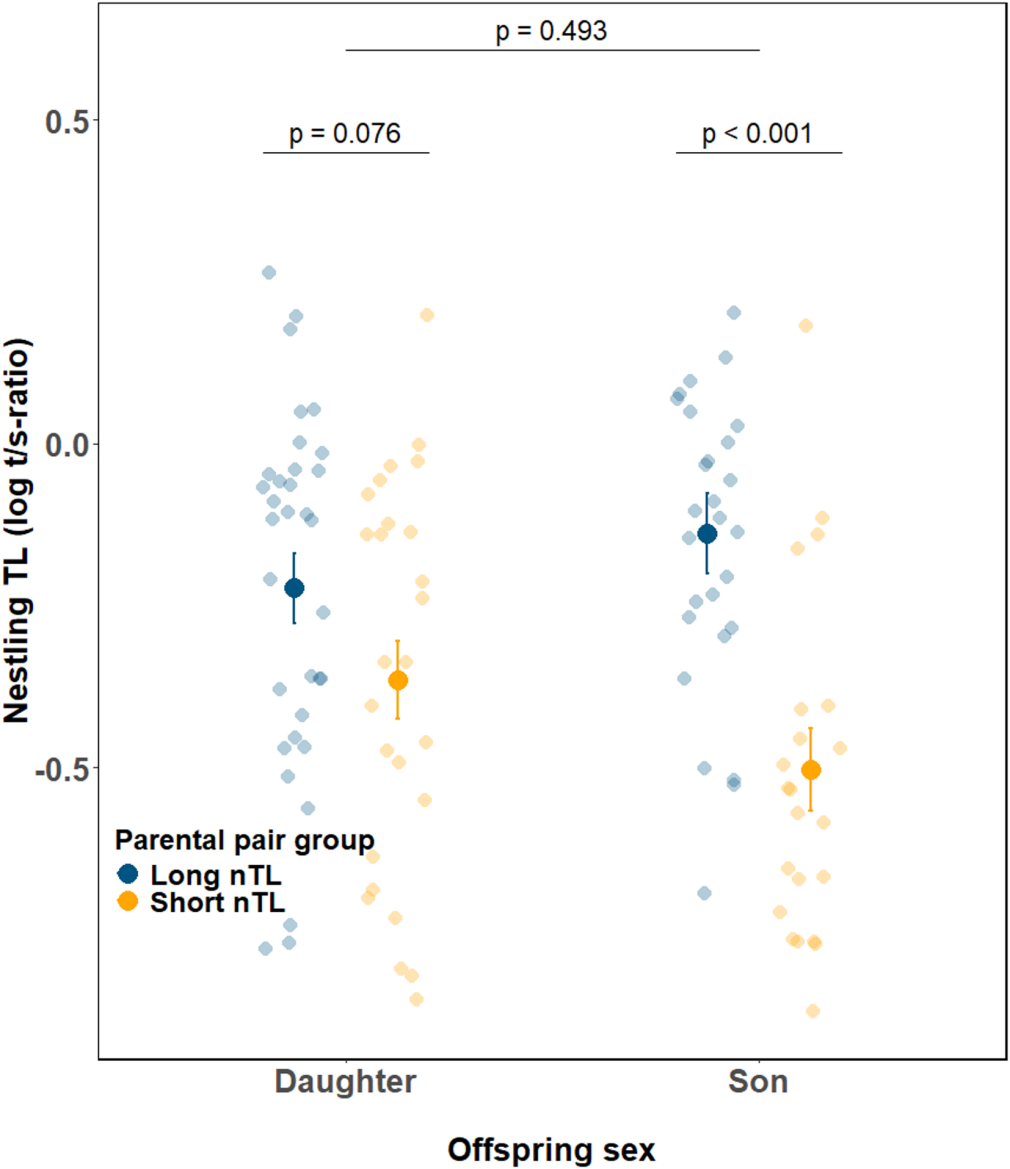
Telomere length (TL) at the nestling stage for sons and daughters from parental pair groups that had either ‘short’ (orange, sons *n* = 22, daughters *n* = 26) or ‘long’ (blue, sons *n* = 27, daughters *n* = 33) TL when nestlings (nTL). Large points and error bars represent mean ± S.E. of log-transformed t/s-ratio values, while semi-transparent small points show raw data. Significant differences between offspring sexes were determined using *post hoc* pairwise comparisons of least square means.

Furthermore, we found that parental pair group affected male and female nestlings differently (parental pair group × nestling sex: F_1,94.0_ = 4.36, *p* = 0.040; Fig. 3, Supporting information Table S3). Specifically, at the nestling stage, sons from parents with long nTL had longer telomeres than sons from parents with short nTL (LS-means ± SE, short vs. long parental pair group, -0.512 ± 0.057 vs. -0.142 ± 0.054; F_1,19.6_ = 22.1, *p* < 0.001; Supporting information Table S6). In contrast, even though daughters showed a similar trend to sons, the effect of parental pair group on female nestlings was marginally non-significant (LS-means ± SE, short vs. long parental pair group, -0.376 ± 0.067 versus − 0.209 ± 0.061, F_1,29.8_ = 3.39 *p* = 0.076; Supporting information Table S7). The average TL of daughters was generally intermediate to that of sons (Fig. 3).

Finally, for embryo TL we also found a trend for an interaction effect between parental age and offspring sex (F_1,94.6_ = 3.25, *p* = 0.074; Supporting information Figure S3; Table S2). Separate analyses for sons and daughters showed that male embryos from old parents tended to have longer telomeres than those from young parents, although this trend was not statistically significant (LS-means ± SE, old vs. young parental group, 0.053 ± 0.052 vs. -0.082 ± 0.051), F_1,27.0_ = 3.41, *p* = 0.076 Supporting information Figure S3; Table S4). In contrast, parental age had no effect on TL in female embryos (old vs. young parental group: -0.058 ± 0.056 vs. -0.060 ± 0.052, F_1,17.9_ = 0.001, *p* = 0.978; Supporting information Figure S3; Table S5).

To corroborate these results and to test if the results would differ if parental TL was included as a continuous variable in the analyses, we also conducted parent-offspring regressions for TL measured at the embryo and nestling stages (see Methods). These analyses largely confirm the results above and we therefore only report them in the Supporting information (Figure S1-S2 and Tables S12-S14).

## Discussion

In this study, our aim was to investigate whether (early-life) TL is determined already during embryonic development (i.e., close to conception), or whether it is critically affected by the processes occurring during the embryonic and early postnatal period. Our study provides evidence for the latter explanation and argues against the idea that individual differences in TL shortly after birth are already set at conception.

Under strict genetic inheritance of TL from parent to offspring, we would have expected a significant difference in embryo TL with respect to the two parental pair groups (pairs where parents had either **short** or **long** nTL) that we had created. Instead, our study suggests that it is the rate of telomere shortening during the embryonic and early postnatal periods that is under control of genetic and/or non-genetic parental effects in zebra finches. Genetic effects that may contribute to the parental-offspring matching in the rate of telomere shortening include the genetic determination of physiological factors affecting e.g., the organization of hematopoietic tissue or of the mechanisms regulating telomere degradation and restoration processes. Parental effects, such as maternal transfer of hormones, antibodies, antioxidants, or parental care (e.g., incubation patterns and nestling feeding effort) may also shape the parental-offspring TL matching at the nestling stage. These parental effects may be genetically based (inherited) investment ‘rules’ for providing care or non-genetic (but still inherited) effects, such as gene methylation patterns or transgenerational antibody transfer^6,7,21,31–33^. Irrespective of the exact mechanisms at work, our results highlight that the late prenatal and the early postnatal developmental stages are critical for regulation of telomere shortening and determining the early post-hatching TL. The latter is an important life history trait as it has been found to predict telomere shortening at adult life stages as well as longevity in a range of species, including in zebra finches^6,8–13,34^.

It is important to keep in mind that in our experiment environmental conditions were kept constant, so purely environmental effects should have been minimal. Thus, under more natural conditions it is certainly possible that exposure to external biotic and/or abiotic factors, such as extreme temperatures or infections, during the sensitive late pre- and early post-hatching period may affect telomere shortening rates (see e.g., Stier et al.^35^) and thus also change the value of nestling TL away from their parents’ nTL value.

Sex differences in TL are thought to contribute to lifespan differences between males and females^31^. How sex-specific TL and telomere shortening rate come about is therefore an important area of research. Our study shows that the effect of the parental pair group on nestling TL was dependent on the sex of the offspring (Fig. 3). Hence, prenatal and early postnatal effects that may have influenced offspring TL appeared to be stronger in sons compared to daughters. This agrees with the ideas of sex-specific parental imprinting of telomere maintenance genes (leading to up- or downregulation of telomere maintenance genes in only one sex) or sex differences in telomere restoration (e.g., through estrogenic control of telomerase) (reviewed in Barrett et al.^32^). It is noteworthy that the effect of the parental pair group was accentuated in male nestlings, so that sons from the **long** nTL parental pair group had the longest telomeres among all nestlings, whereas the sons from the **short** nTL parental pair group had the shortest telomeres (Fig. 3).

In birds, it seems that inheritance of TL is characterized by a stronger mother-offspring relationship and weaker (or even sometimes absent) relationship between father and offspring^11,35,36^. It is possible that by our experimental design (i.e., matching pairs depending on their TL as nestlings), we may have created pairs that differed in parental investment, and this resulted in different telomere trajectories for male and female offspring. Romero-Haro et al. ^37^ have recently shown that male Japanese quail chicks from lines selected for high egg investment had longer telomeres than those from low egg investment lines but with no difference among female chicks. In zebra finches, females have been found to be more sensitive to nutritional stress and increased within-brood competition compared to males^38–40^. Moreover, Noguera et al.^6^ have shown that zebra finch female, but not male, offspring experience reduced telomere shortening when supplied with high micronutrient diet. Thus, one might expect daughters to be more sensitive to telomere shortening. However, another study by Marasco et al.^29^ showed that young zebra finch females housed under unpredictable food conditions reared sons with shorter TL compared to those reared by females under controlled conditions. This effect was absent in daughters.

In our study, we found no difference in clutch size or brood size at hatching that would suggest differences in egg investment between the two parental pair groups (see Supporting Information Table S11 for details). Moreover, hatching success did not differ between parental pair groups or parental age groups (Supporting Information Table S10). There was a lower proportion of eggs that developed embryonic tissue in the first breeding attempt (when we collected embryos) compared to the second breeding attempt (when parents were raising nestlings). This is not unexpected as captive zebra finches breeding for the first time in life often have lower hatching success compared to later breeding attempts (own personal observation). This may in part be due to lower male fertility at first breeding. It has been shown that in four captive populations of zebra finches male fertility significantly increased over their first three clutches in life and stayed high afterwards^41^. However, we have no reason to believe that this effect would have been different between parental pair groups since they did not differ in breeding experience (see Methods). Indeed, the proportion of infertile eggs did not differ between parental pair groups (*p* > 0.5, Table S11). Parental age groups differed in the mean TL at nestling age (i.e., in nTL). Even though this is a potentially interesting result (and may reflect disappearance of individuals with short nTL at an earlier chronological age, see for example)^10^, the key results of our study were consistent in both parental age groups. It should therefore not affect the main results and conclusions of this study. Breeding pairs and offspring were held under constant and benign housing conditions, and it therefore seems less likely that the sex differences in nestling TL are due to variation in housing condition-related stressors influencing early development. However, there may still have been differences in investment of egg size, egg components, parental incubation effort or feeding behavior that affected the sexes differently. Finally, the results from our study suggest that sex-specific effects on TL may be subtle, and act already at very early stages of development. These effects might, thus, be masked in studies investigating later stages of development or adult life stages.

Several longitudinal studies on birds show that the age of parents and grand-parents can affect offspring TL negatively^29,42,43^ or positively^44^. In our study, we found that parental age group (age cohort) had no effect on nestling TL. Although male embryos produced by old parents tended to have longer telomeres than those produced by young parents, this effect fades away at the nestling stage. Marasco et al.^29^ have previously found that both male and female zebra finch offspring from older parents had shorter TL, which contrasts with our study. However, the age difference between young and old parents was considerably larger in Marasco et al.’s study compared to ours (one versus three years difference in age) which might explain the discrepancy.

We acknowledge that there are caveats to our study. Due to the nature of the experimental design, embryo and nestling measures were based on different individuals that came from subsequent clutches of the same pair. Moreover, because eggs of the first clutch were collected immediately and incubated in an incubator and eggs from the second clutch were naturally incubated, different clutches were exposed to somewhat different incubation regimes. However, Noguera et al.^6^ analyzed the effect of clutch number and within-clutch laying order on embryonic and postnatal TL in first-time breeding zebra finches (i.e., similarly inexperienced birds as in our experiment). They found that TL (in embryos) increased by 11% from the first to the second clutch and that within-clutch TL patterns found for embryos were similar to those found in nestlings, juveniles and adults^6^. Moreover, in that study, effects of laying order were the same irrespective of clutch size or number. This suggests that investment patterns across subsequent clutches are fairly stable and not affected by different experimental incubation regime (incubator vs. parental incubation). We found no effect of parental pair group (short vs. long nTL) on hatching success, nestling body mass or early life mortality (up to day 10; for details see Supporting Information Table S9 and S10). Moreover, we would also expect more variation in TL measured in nestlings compared to embryos since the former likely experienced more variable incubation conditions. Thus, if anything, differences in embryo TL should be more pronounced compared to nestling TL.

In conclusion, results from our TL assortative pairing experiment show that nestling TL, but not embryo TL, matches parental nTL. This suggests that telomere shortening during early prenatal and postnatal development shows similar patterns in parents and offspring, but that TL itself is not set at conception. Moreover, even though there may be no general sex difference in TL at birth, our study suggests that there may be different mechanisms, or similar mechanisms but with sex-dependent effects on TL that differ in magnitude between males and females shortly after birth. Our results support ‘adaptive regulation models’^45^, such as the ‘fetal programming of telomere biology hypothesis’^16^, which proposes that conditions experienced during early development (prenatal and early postnatal), such as parental quality, nutrition and environmental stress, can shape the initial setting and subsequent dynamics of early-life telomere length, potentially also affecting health and lifespan later in life. In contrast, our findings argue against the idea that TL is primarily genetically determined at—or shortly after—conception, and that telomere trajectories follow parallel paths across individuals over their (entire) lives^7,46^. Future studies are needed to determine how these differences in TL are affected by maternal and paternal contributions and how the differences are carried over into adulthood.

## Methods

### Study population

We used 251 captive adult zebra finches (*Taeniopygia guttata*) of known age and with nTL (i.e., nestling TL) measured ca. 10 days after hatching (range 10–13 days). The nTL of these adults was used to select pairs for the breeding experiment (see below). Pairs were housed indoors in individual breeding cages (59 × 33 × 57 cm). Temperature (22 ± 2 °C) and photo period (12 L: 12D) were maintained constant. Birds had *ad libitum* access to a mixed seed diet (Franks Zoofor AB), drinking water, egg food and sepia shells.

### Selection of breeding pairs

To obtain a sufficient number of breeding pairs and offspring, we used zebra finches that came from two different cohorts: a cohort with ‘young’ birds (mean age ± SE = 8.2 ± 0.3 months (range 6–9 months), *n* = 106) and a cohort with ‘old’ birds (26.8 ± 0.1 months (range 25–33 months), *n* = 145). From the young cohort of birds, we selected the individuals that fell within the top and bottom 35% of this cohort’s nTL distribution, and from the older cohort, we selected the individuals that fell within the top and bottom 32% of the nTL distribution of this cohort (a slightly higher percentage was chosen for the younger birds since we had fewer birds in this group). We call birds from the top 32–35% of the nTL distribution the ‘**long**’ parental pair group, and birds from the bottom 32–35% of the nTL distribution the ‘**short**’ parental pair group. Our selection of birds resulted in 34 males and 36 females from the young cohort, and 49 males and 44 females from the old cohort available for breeding. Because not all birds paired with their partners and started breeding, we ended up with a total number of 22 pairs from the young cohort (12 with long and 10 with short nTL) and 19 pairs from the old cohort (9 with long and 10 with short nTL). The parents’ nTL differed significantly between the two parental pair groups (young cohort (LS-mean ± SE of log-transformed T/S ratio values): short nTL group (-0.878 ± 0.049) versus long nTL group (-0.323 ± 0.049): F_1,60_ = 64.07, *p* < 0.001; old cohort: short nTL group (-0.597 ± 0.029) versus long nTL group (-0.134 ± 0.032): F_1,55_ = 111.68, *p* < 0.001). To avoid inbreeding effects, we did not pair siblings/half-siblings or cousins with each other. The experimenters were not aware of experimental groupings when collecting samples.

### Breeding experiments

Most of the adult birds (93%, *n* = 138) used in this experiment were first-time breeders. We used the same protocol as Noguera et al.^6^, collecting all the eggs of the first clutch for embryo analyses and then immediately allowing the pairs to lay a new clutch that they incubated and raised. The two parental age groups (i.e., **young** and **old**) were set up for breeding during summer 2020, with the young group starting about one month before the old. After pairs were formed, we made daily checks to monitor the breeding process (nest building, start of egg laying, etc.). To stimulate the birds to breed, lettuce leaves and/or cucumber slices were given every 2–3 days, and water was sprayed on the breeding cages every 1–2 days. A week after the birds were paired, a cardboard nest box (13 × 13 × 11.5 cm) was attached at the side of each breeding cage. Hay, coconut fiber and cotton wool were given as nesting material. We recorded the breeding stage (including start of nest building) for each nest box daily.

We collected the first clutch of each pair to obtain measures of embryo TL. Each egg was collected on the same day it was laid and replaced with a dummy egg. The collected eggs were stored at room temperature (24–25 °C) until the completion of the clutch. The whole clutch of a pair was then moved into an incubator (Ruvmax, Ödskölt, Sweden) with constant temperature (37.7 ± 0.5 °C), and relative ambient humidity (55 ± 5%). After 6 days in the incubator, eggs were checked for embryo growth with a flashlight and all eggs that had developed embryo tissue (*n* = 110) were frozen and stored until further analysis in the lab.

We then allowed pairs to start laying a new clutch by removing the dummy eggs. Once the incubation of the new clutch had started, we stopped the daily checks to minimize disturbance but checked for unfertilized eggs by candling with a flashlight five days after the start of incubation. Some pairs incubated unfertilized eggs (*n* = 5 pairs), or the clutch was unsuccessful for other reasons (e.g., unhatched fertilized eggs, *n* = 4 pairs), in which cases we removed the eggs, and the pairs were allowed to lay again. Nests with fertilized eggs were checked for hatching after another six days (until day 11 after the start of incubation). When the eggs started hatching, we checked the nest daily and individually color-marked the dune feathers of the hatchlings (Q-connect, liquid ink highlighter). A blood sample (20 µl) and body mass measurement were collected on day 10 post-hatching, when most nestlings had reached a body mass of ≥ 5 g (*n* = 96). A 0.4 mm needle (Henke-Ject, lot: 14-14575) was used to puncture the wing vein of each nestling. Blood was collected using a 20 µl microcapillary tube (Drummond, Capillary Tubes Glass, 20 µl volume, 64 mm length, Non-Sterile Microcaps^®^), stored in lithium heparin-coated tubes (Eppendorf) for a maximum of two hours before being frozen at -40 °C until further analysis in the lab. For nestlings weighing < 5 g on day 10, blood sampling was delayed until they reached a body mass of ≥ 5 g (day 11: *n* = 6; day 12: *n* = 5; day 13: *n* = 1). There were no differences in the sampling age of nestlings between parental pair groups, parental age groups or offspring sex (all *p* > 0.10; see Supporting Information Table S9). The same blood sampling method was applied to parent birds when they were sampled as nestlings.

Nestling body mass on day 10 after hatching, hatching rate, and mortality rate during the first 10 days of life did not differ between parental pair groups or parental age groups (all *p* > 0.10; for details see Supporting Information Table S9 and S10). In addition, clutch size showed no significant differences between clutch number (first and second clutch), parental pair groups, or parental age groups (all *p* > 0.10; for details see Supporting Information Table S11).

### Ethical statement

Housing, experiments, and sampling of zebra finches were performed under permits from the Malmö/Lund ethical committee (permit numbers 5.2.18–8479/17 and 5.8.18–16951/2018). All experimental methods were carried out in accordance with national guidelines and regulations. The methods are reported in accordance with the ARRIVE guidelines.

### DNA extraction

Blood samples and embryo tissue samples were extracted with DNA extraction kits from Macherey-Nagel. For the measurement of nTL of the parents, we used NucleoSpin^®^ Blood QuickPure kit. For measurement of offspring TL at the embryo and nestling stages, we used NucleoSpin^®^ Tissue kit. We used the same extraction kit for the embryo and nestling blood samples so that they could be directly compared, as the type of extraction (kit) could potentially influence measurements of TL ^47,48^. Extractions were made according to the manufacturer’s protocols, and concentration and purity of DNA was examined with NanoDrop™ 2000 Spectrophotometers (Thermo Fisher Scientific). Extracted DNA was diluted in an elution buffer (5 nM Tris/HCL, pH 8.5) and kept at -20 °C for short-term storage, and at -40 °C for long-term storage.

### qPCR and qPCR data normalization

To measure TL, we used the qPCR protocol described in Criscuolo et al.^49^ with some modifications (described below). The control single copy gene glyceraldehyde-3-phosphate dehydrogenase (GAPDH) was amplified using the primers GAPDH-F (5′-GACCTGCCGTCTGGAAAAAC − 3′) and GAPDH-R (5′-CCTGGTCCTCTGTGTATGCC − 3′). These primers were designed by us and are specific to the zebra finch GAPDH (GenBank ID: NM_001198610). We used the same telomere primers as Criscuolo et al.^49^: Tel1b (5′-CGGTTTGTTTGGGTTTGGGTTTGGGTTTGGGTTTGGGTT-3′) and Tel2b (5′-GGCTTGCCTTACCCTTACCCTTACCCTTACCCTTACCCT-3′). We used 25 µl reactions for both GAPDH and Telomere runs, with 12.5 µl Platinum™ SYBR™ Green qPCR SuperMix-UDG (Invitrogen, Thermo Fisher Scientific), 0.1 µl Rox and 5 µl of DNA (1ng/µl) per reaction. For the telomere reactions, we used 0.5 µl of each primer (10nM) per reaction. For the GAPDH primers, we used 1 µl of each primer (10nM). We run Telomere reactions for 25 cycles, and GAPDH reactions for 35 cycles. qPCR-reactions for both GAPDH and Telomere were performed in triplicates for each sample. Telomere and GAPDH runs were performed on separate plates, using Skirted Hard-Shell^®^ 96-Well PCR Plates (Bio-Rad, model.id: HSP9601). qPCRs were performed in a CFX96™ Realtime System C1000 Touch^®^ Thermal Cycler (Bio-Rad). Thermal cycling conditions for Telomere reactions were 10 min at 95 °C followed by 25 cycles of 15 s at 95 °C, 30 s at 58 °C and 30 s at 72 °C. The GAPDH thermal cycle started with 10 min at 95 °C followed by 35 cycles of 15 s at 95 °C, 1 min at 60 °C and 30 s at 72 °C. A melting curve analysis was performed to check amplificons specificity, followed immediately by heating at 95 °C for 1 min, cooling to 58 °C for 30 s, and then gradually increasing the temperature to 95 °C at a rate of 0.5 °C per second while recording fluorescence. Every plate included a 2-fold standard dilution series with 5 dilution points (from 4 ng/µl to 0.25 ng/µl) and one reference sample (i.e., a ‘golden sample’ that was included on all plates) for calibration between plates.

The outputs of the qPCR-plates were examined using CFX Maestro Software (Bio-Rad). We checked the quality of each plate with the software’s built-in quality control. The criteria for successful qPCR-plates were: (i) efficiency = 100 ± 15 (the actual range was 91% − 114.5%); (ii) standard curve r^2^ > 0.98; (iii) standard deviation (SD, intra-assay repeatability) of Ct-value among sample triplicates < 0.2; (iv) no contamination in the no template control; (v) melting curves of qPCR product matched the melting curve of the target fragments. Samples, or plates, that did not meet the criteria described above were ran again, until all criteria were met.

To further reduce the effect of measurement errors (see^50^) all samples were analyzed twice in separate qPCR runs conducted approximately one year apart. We applied slightly different methods of sample randomization between the two runs. For the first run, nestling and embryo DNA samples were completely randomized across plates. For the second run, we kept embryo and nestling samples from the same parents on the same plate but randomized among nests (while still including samples from both parental pair groups and parental age groups on each plate).

We took the mean Ct-value of the triplicates for telomere and GAPDH, respectively, and then calculated relative TL (t/s-ratio) according to Pfaffl’s method^51,52^. First, we calculated ΔCt (ΔCt = Ct_telomere – Ct_control) to represent the difference in amplification cycles between the telomere region and the control gene of an unknown sample. We also calculated ΔCt for a reference sample (the “golden sample”) included on every plate. Next, we calculated ΔΔCt (ΔΔCt = ΔCt_unknown sample – ΔCt_reference) to quantify the relative abundance of telomeric DNA compared to the reference. Finally, t/s-ratios were calculated using the formula t/s = 2^(-ΔΔCt), assuming optimal and identical real-time amplification efficiencies for the telomere and GAPDH reaction. This provided the relative telomere length of each unknown sample in comparison to the reference sample. The same individual was used as the “golden sample” for both runs.

Results from the two qPCR runs (performed one year apart using a new 1 ng/µl working dilution prepared from the same DNA extraction) showed good agreement. Inter-assay reproducibility, assessed as the intraclass correlation coefficient (ICC) calculated from a linear mixed-effects model on the log-transformed t/s-ratios with individual ID as a random factor, was 0.662 (SE = 0.038; 95% CI = 0.585–0.732; *p* < 0.001). In addition, the log-transformed t/s-ratios from the two runs were strongly correlated (Pearson’s *r* = 0.71, *n* = 218, *p* < 0.001). For each individual, we used the mean of log-transformed t/s-ratio value of the two qPCR runs as the estimate of telomere length, which we later used in all the statistical analyses.

### Molecular sexing

Molecular sexing of embryos, as well as of nestlings that died before they could be sexed based on their plumage, was done with PCR^53^, using the primers P2 (5′-TCTGCATCGCTAAATCCTTT-3′) and P8 (5′-CTCCCAAGGATGAGRAAYTG-3′). In a 10 µl reaction, we used 5 µl multiplex PCR master mix (Qiagen), 1 µl of DNA (conc. ~10 ng/µl), primers P2/P8 (at a concentration of 200 nM) and ddH_2_O. Thermal cycling conditions were: 15 min at 95° C, followed by 40 cycles of (30 s at 95 °C, 45 s at 50 °C and 45 s at 72 °C), and finally 3 min at 72 °C. Sexing was obtained by running the final PCR products through gel electrophoresis: 3 µl PCR product was mixed with 2 µl stopmix (0.25% Bromophenol blue, 0.25% Xylene Cyanole, 15% Ficoll in 0.5 M EDTA solution) and electrophoresis was performed on 2% agarose gel, starting with 10 min at 70 V for heating up, and then 50 min at 85 V.

### Statistical analyses

Statistical analyses were performed using R 4.3.1^54^. To test the effects of developmental stage, parental pair group (short vs. long nTL) and parental age group on offspring TL, we ran linear mixed models (LMM) using the *lme4* package (version 1.1.34)^55^ and the *lmerTest* package^56^. The t/s-ratio values were log-transformed to normalize the distribution first. We then used the full dataset, setting mean log-transformed t/s-ratio value from the two measurement runs as the dependent variable, and developmental stage (embryo or nestling), parental pair group (long or short nTL), parental age group (young or old cohort) and offspring sex (daughter or son) as fixed factors. We also included “cage” as a random factor to account for common rearing environment and relatedness among siblings. Post-hoc comparisons between specific groups were done using the package “*emmeans*” and are presented as least-squares means, adjusted with Dunn-Sidak correction for pair-wise comparisons^57^. Residuals of all models were checked for normality. The significance level was set at alpha ≤ 0.05. We also calculated the effect size (η^2^) for both fixed factors and interactions using the package “*sjstats*”^58^.

To disentangle the different effects of the parental pair group on offspring TL at the two developmental stages, we ran separate LMMs for the embryo stage dataset and the offspring nestling stage dataset. In these analyses, we included mean log-transformed t/s ratio value as the dependent variable, parental pair group, parental age group and offspring sex as fixed factors, and cage of offspring as random factors. Furthermore, we ran LMMs for female and male offspring separately, to analyze if parental pair group and parental age group had different effects on TL in sons and daughters. In the nestling model, we also included body mass as an additional covariate. For all of the above, we present the results from the reduced models in which non-significant interactions (*p* > 0.1) were backward eliminated. For transparency and because removal of non-significant terms is known to increase type I errors^59^, we also present the full models that include all fixed factors and their two- and three-way interactions in the Supporting Information. Overall, results from the reduced and the full models were qualitatively very similar. In our experimental set-up, we categorize parents in two groups (long and short) based on their nestling TL, creating distinct groups of pairs that differ in nTL. However, because categorization of a continuous variable such a TL may be problematic^60^, we also analyzed the same dataset using linear mixed models using offspring TL as a dependent variable and developmental stage, offspring sex, mid-parent age (mean age of father and mother in days), mid-parent nTL (average nestling TL of father and mother) and their interactions as predictor variables. The results from the continuous models largely confirm the results of the categorical models and we therefore only report them in the Supporting information. The data generated or analyzed during this study is available at: (https://figshare.com/s/ca01f5d953726e7e7088).

## Supplementary Information

Below is the link to the electronic supplementary material.

Supplementary Material 1

## Data Availability

The data generated or analyzed during this study is available at: (https://figshare.com/s/ca01f5d953726e7e7088).
