# Supplementary material for: Assortative breeding experiment in a songbird suggests telomere length is determined during early life rather than at conception
Source: Sci Rep. 2025 Oct 21;15:36510. doi: 10.1038/s41598-025-23517-7 (PMC12540821; doi:10.1038/s41598-025-23517-7)
Supplement: Supplementary file 1 — Supplementary Material 1 [file 41598_2025_23517_MOESM1_ESM.docx]

**Supporting material**

***Table S1.*** **Reduced model** showing the results of a linear mixed model (LMM) testing the dependence of ***offspring telomere length*** (TL; log-transformed t/s-ratio) on development stage (embryo or nestling), parental pair group (long or short nTL), parental age group (young or old), and offspring sex (son or daughter), as well as the interactions where p < 0.10. Significant factors are marked in bold (**p < 0.05**). nTL = the parents’ TL when they were nestlings (i.e., 10–13 days after hatching). See below for full model.

| ***Fixed effects*** | *F* | *Df* | | *p* | | *Eta2 (η^2^) (partial)* |
| --- | --- | --- | --- | --- | --- | --- |
| **Development stage** | **63.62** | **1, 205.2** | | **< 0.001 ***** | | **0.24** |
| **Parental pair group** | **13.23** | **1, 32.5** | | **< 0.001 ***** | | **0.29** |
| Parental age group | 1.72 | 1, 33.1 | | 0.199 | | 0.05 |
| Offspring sex | 0.21 | 1, 206.9 | | 0.650 | | < 0.001 |
| **Development stage × Parental pair group** | **6.51** | **1, 201.7** | | **0.011 *** | | **0.03** |
| ***Random effects*** | *Variance* | *Std.Dev* | *# group* | | *# observations* | |
| Cage | 0.007 | 0.082 | 41 | | 218 | |
| Residuals | 0.057 | 0.238 |  | |  | |

**Full model**. Same as above but including all two-way and three-way interactions.

| ***Fixed effects*** | *F* | *Df* | | *p* | | *Eta2 (η^2^) (partial)* |
| --- | --- | --- | --- | --- | --- | --- |
| **Development stage** | **57.89** | **1, 197.4** | | **< 0.001** | | **0.23** |
| **Parental pair group** | **14.33** | **1, 30.5** | | **< 0.001** | | **0.31** |
| Parental age group | 1.73 | 1, 30.8 | | 0.197 | | 0.05 |
| Offspring sex | 0.06 | 1, 199.8 | | 0.812 | | < 0.001 |
| **Development stage × Parental pair group** | **7.22** | **1, 197.1** | | **0.008** | | **0.04** |
| Development stage × Parental age group | 0.01 | 1, 197.6 | | 0.908 | | < 0.001 |
| Development stage × Offspring sex | 1.87 | 1, 196.9 | | 0.172 | | < 0.001 |
| Parental pair group × Parental age group | 0.91 | 1, 30.7 | | 0.348 | | 0.03 |
| Parental pair group × Offspring Sex | 1.65 | 1, 201.0 | | 0.200 | | < 0.001 |
| Parental age group × Offspring sex | 2.80 | 1, 201.0 | | 0.096 | | 0.01 |
| Development stage × Parental pair group × Parental age group | 0.70 | 1, 197.0 | | 0.404 | | < 0.001 |
| Development stage × Parental pair group × Offspring sex | 3.36 | 1, 197.9 | | 0.068 | | 0.02 |
| Development stage × Parental age group × Offspring sex | 0.01 | 1, 197.7 | | 0.910 | | < 0.001 |
| Parental pair group × Parental age × Offspring sex | 0.89 | 1, 201.0 | | 0.347 | | < 0.001 |
| ***Random effects*** | *Variance* | *Std.Dev* | *# group* | | *# observations* | |
| Cage | 0.006 | 0.075 | 41 | | 218 | |
| Residuals | 0.057 | 0.238 |  | |  | |
|  |  |  |  | |  | |

***Table S2.*** **Reduced model** showing the results of a linear mixed model (LMM) testing the dependence of ***embryo telomere length*** (TL; log-transformed t/s-ratio) on parental pair group (long or short nTL), parental age group (young or old), and embryo sex, as well as the interactions where p < 0.10. Significant factors are marked in bold (**p < 0.05**). nTL = the parents’ TL when they were nestlings (i.e., 10–13 days after hatching). See below for full model.

| ***Fixed effects*** | *F* | *df* | | *p* | | *Eta2 (η^2^) (partial)* | |
| --- | --- | --- | --- | --- | --- | --- | --- |
| Parental pair group | 0.37 | 1, 31.7 | | 0.546 | | 0.01 | |
| Parental age group | 1.24 | 1, 31.4 | | 0.274 | | 0.04 | |
| Embryo sex | 0.89 | 1, 95.7 | | 0.349 | | 0.01 | |
| Parental age group × Embryo sex | 3.25 | 1, 94.6 | | 0.075 | | 0.03 | |
| ***Random effects*** | *Variance* | | *Std.Dev* | | *# group* | | *# observations* |
| Cage | 0.018 | | 0.135 | | 36 | | 110 |
| Residuals | 0.034 | | 0.186 | |  | |  |

**Full model.** Same as above but including all two- and three-way interactions.

|  |  |  |  |  |  |  |  |
| --- | --- | --- | --- | --- | --- | --- | --- |
| \| ***Fixed effects*** \| *F* \| *df* \| \| *p* \| \| *Eta2 (η^2^) (partial)* \| \| \| --- \| --- \| --- \| --- \| --- \| --- \| --- \| --- \| \| Parental pair group \| 0.48 \| 1, 29.0 \| \| 0.494 \| \| 0.02 \| \| \| Parental age group \| 1.11 \| 1, 29.0 \| \| 0.300 \| \| 0.04 \| \| \| Embryo sex \| 1.58 \| 1, 95.6 \| \| 0.211 \| \| 0.02 \| \| \| Parental pair group × Parental age group \| 0.61 \| 1, 29.0 \| \| 0.443 \| \| 0.02 \| \| \| Parental pair group × Embryo sex \| 1.08 \| 1, 95.6 \| \| 0.301 \| \| 0.01 \| \| \| **Parental age group × Embryo sex** \| **4.30** \| **1, 95.6** \| \| **0.041*** \| \| **0.04** \| \| \| Parental pair group × Parental age group × Embryo sex \| 0.92 \| 1, 95.6 \| \| 0.340 \| \| < 0.01 \| \| \| ***Random effects*** \| *Variance* \| \| *Std.Dev* \| \| *# group* \| \| *# observations* \| \| Cage \| 0.019 \| \| 0.138 \| \| 36 \| \| 110 \| \| Residuals \| 0.034 \| \| 0.185 \| \|  \| \|  \| \|  \|  \| \|  \| \|  \| \|  \| |  |  |  |  |  |  |  |

***Table S3.*** **Reduced model** showing the results of a linear mixed model (LMM) testing the dependence of ***nestling telomere length*** (TL; log-transformed t/s-ratio) on parental pair group (long or short nTL), parental age group (young or old), and offspring sex, as well as the interactions where p < 0.10. Significant factors are marked in bold (**p < 0.05**). nTL = the parents’ TL when they were nestlings (i.e., 10–13 days after hatching). See below for results when testing the full model.

|  |  |  |  | |  | | |  | | |  |  | |
| --- | --- | --- | --- | --- | --- | --- | --- | --- | --- | --- | --- | --- | --- |
| ***Fixed effects*** | | | | | *F* | | *df* | *P* | | *Eta2 (η^2^) (partial)* | | | |
| **Parental pair group** | | | | | **13.817** | | **1, 34.835** | **< 0.001 ***** | | **0.28** | | | |
| Parental age group | | | | | 0.450 | | 1, 34.213 | 0.507 | | 0.01 | | | |
| Nestling sex | | | | | 0.485 | | 1, 92.810 | 0.488 | | < 0.01 | | | |
| Body mass at day10 | | | | | 1.308 | | 1, 101.435 | 0.255 | | 0.01 | | | |
| **Parental pair group × Nestling sex** | | | | | **4.355** | | **1, 93.998** | **0.040 *** | | **0.04** | | | |
| ***Random effects*** | | | | | *Variance* | | *Std.Dev* | *# group* | | | *# observations* | | |
| Cage | | | | | 0.021 | | 0.146 | 41 | | | 108 | | |
| Residuals | | | | | 0.052 | | 0.228 |  | | |  | | |
|  | | | | |  | |  |  | | |  | | |

**Full model.** Same as above but including all two- and three-way interactions.

| ***Fixed effects*** | *F* | *df* | *P* | *Eta2 (η^2^) (partial)* | |
| --- | --- | --- | --- | --- | --- |
| **Parental pair group** | **13.236** | **1, 34.471** | **< 0.001***** | **0.28** | |
| Parental age group | 0.538 | 1, 34.267 | 0.468 | 0.02 | |
| Nestling sex | 0.505 | 1, 90.942 | 0.479 | < 0.01 | |
| Body mass at day10 | 1.111 | 1, 98.770 | 0.294 | 0.01 | |
| Parental pair group × Parental age group | 0.168 | 1, 34.336 | 0.684 | < 0.01 | |
| **Parental pair group × Nestling sex** | **5.383** | **1, 92.055** | **0.023*** | **0.06** | |
| Parental age group × Nestling sex | 2.261 | 1, 91.857 | 0.136 | 0.02 | |
| Parental pair group × Parental age group × Nestling sex | 0.575 | 1, 92.747 | 0.450 | < 0.01 | |
| ***Random effects*** | *Variance* | *Std.Dev* | *# group* | | *# observations* |
| Cage | 0.025 | 0.157 | 41 | | 108 |
| Residuals | 0.051 | 0.225 |  | |  |
|  |  |  |  | |  |

***Table S4.*** Determinants of telomere length (TL) in sons at the embryo stage. **Reduced model** showing the results of a linear mixed model (LMM) testing the dependence of the ***sons’ embryo stage TL*** (log-transformed t/s-ratio) on parental pair group (long or short nTL) and parental age group (young or old), as well as the interactions where p < 0.10. Significant factors are marked in bold (**p < 0.05**). nTL = the parents’ TL when they were nestlings (i.e., 10–13 days after hatching). See below for full model.

| ***Fixed effects*** | *F* | *df* | | | *p* | *Eta2 (η^2^) (partial)* | |
| --- | --- | --- | --- | --- | --- | --- | --- |
| Parental pair group | 0.022 | 1, 27.061 | | | 0.884 | < 0.01 | |
| Parental age group | 3.41 | 1, 26.979 | | | 0.076 | 0.11 | |
| ***Random effects*** | *Variance* | | *Std.Dev* | *# group* | | | *# observations* |
| Cage | 0.015 | | 0.122 | 30 | | | 58 |
| Residuals | 0.038 | | 0.195 |  | | |  |
|  |  | |  |  | | |  |

**Full model**. Same as above but including all two-way and three-way interactions

| ***Fixed effects*** | *F* | *df* | | | *p* | *Eta2 (η^2^) (partial)* | |
| --- | --- | --- | --- | --- | --- | --- | --- |
| Parental pair group | 0.02 | 1, 26.06 | | | 0.892 | < 0.01 | |
| Parental age group | 3.22 | 1, 26.06 | | | 0.084 | 0.11 | |
| Parental pair group × Parental age group | 0.03 | 1, 26.06 | | | 0.875 | < 0.01 | |
| ***Random effects*** | *Variance* | | *Std.Dev* | *# group* | | | *# observations* |
| Cage | 0.016 | | 0.127 | 30 | | | 58 |
| Residuals | 0.038 | | 0.195 |  | | |  |
|  |  | |  |  | | |  |

***Table S5.*** Determinants of telomere length (TL) in daughters at the embryo stage. **Reduced model** showing the results of a linear mixed model (LMM) testing the dependence of the ***daughters’ embryo stage TL*** (log-transformed t/s-ratio) on parental pair group (long or short nTL) and parental age group (young or old), as well as the interactions where p < 0.10. Significant factors are marked in bold (**p < 0.05**). nTL = the parents’ TL when they were nestlings (i.e., 10–13 days after hatching). See below for results when testing the full model.

| ***Fixed effects*** | *F* | *df* | | *p* | | *Eta2 (η^2^) (partial)* | |
| --- | --- | --- | --- | --- | --- | --- | --- |
| Parental pair group | 0.632 | 1,17.804 | | 0.437 | | 0.03 | |
| Parental age group | 0.001 | 1,17.918 | | 0.978 | | < 0.01 | |
| ***Random effects*** | *Variance* | | *Std.Dev* | | *# group* | | *# observations* |
| Cage | 0.02 | | 0.141 | | 29 | | 52 |
| Residuals | 0.035 | | 0.186 | |  | |  |
|  |  | |  | |  | |  |

**Full model**. Same as above but including all two-way and three-way interactions.

| ***Fixed effects*** | *F* | *df* | | *p* | | *Eta2 (η^2^) (partial)* | |
| --- | --- | --- | --- | --- | --- | --- | --- |
| Parental pair group | 0.92 | 1, 13.61 | | 0.353 | | 0.06 | |
| Parental age group | 0.00 | 1, 13.61 | | 0.955 | | < 0.01 | |
| Parental pair group × Parental age group | 2.17 | 1, 13.61 | | 0.164 | | 0.14 | |
| ***Random effects*** | *Variance* | | *Std.Dev* | | *# group* | | *# observations* |
| Cage | 0.015 | | 0.121 | | 29 | | 52 |
| Residuals | 0.037 | | 0.192 | |  | |  |
|  |  | |  | |  | |  |

***Table S6.*** Determinants of telomere length (TL) in sons at the nestling stage. **Reduced model** showing the results of a linear mixed model (LMM) testing the dependence of the ***sons’ nestling stage TL*** (log-transformed t/s-ratio) on parental pair group (long or short nTL), parental age group (young or old), and offspring body mass at day10, as well as the interactions where p < 0.10. Significant factors are marked in bold (**p < 0.05**). nTL = the parents’ TL when they were nestlings (i.e., 10–13 days after hatching). See below for full model.

| ***Fixed effects*** | *F* | *df* | *p* | *Eta2 (η^2^) (partial)* |
| --- | --- | --- | --- | --- |
| **Parental pair group** | **22.096** | **1, 19.598** | **< 0.001 ***** | **0.53** |
| Parental age group | 1.633 | 1, 20.407 | 0.216 | 0.07 |
| Body mass at day10 | 2.278 | 1, 44.999 | 0.138 | 0.05 |
| ***Random effects*** | *Variance* | *Std.Dev* | *# group* | *# observations* |
| Cage | 0.020 | 0.136 | 31 | 49 |
| Residuals | 0.039 | 0.197 |  |  |
|  |  |  |  |  |

**Full model**. Same as above but including all two-way and three-way interactions.

|  | |  | |  | |  | |  | |  | |  |
| --- | --- | --- | --- | --- | --- | --- | --- | --- | --- | --- | --- | --- |
| ***Fixed effects*** | | *F* | | *df* | | *p* | | *Eta2 (η^2^) (partial)* | |  |  |  |
| **Parental pair group** | | **20.058** | | **1, 19.800** | | **< 0.001 ***** | | **0.50** | |  |  |  |
| Parental age group | | 1.546 | | 1, 20.373 | | 0.228 | | 0.07 | |  |  |  |
| Body mass at day10 | | 2.277 | | 1, 44.000 | | 0.138 | | 0.05 | |  |  |  |
| Parental pair group × Parental age group | | 0.055 | | 1, 19.822 | | 0.817 | | < 0.01 | |  |  |  |
| ***Random effects*** | | *Variance* | | *Std.Dev* | | *# group* | | *# observations* | |  |  |  |
| Cage | | 0.021 | | 0.146 | | 31 | | 49 | |  |  |  |
| Residuals | | 0.038 | | 0.195 | |  | |  | |  |  |  |
|  | |  | |  | |  | |  | |  |  |  |

***Table S7.*** Determinants of telomere length (TL) in daughters at the nestling stage. **Reduced model** showing the results of a linear mixed model (LMM) testing the dependence of the ***daughters’ nestling stage TL*** (log-transformed t/s-ratio) on parental pair group (long or short nTL), parental age group (young or old), and offspring body mass at day10, as well as the interactions where p < 0.10. Significant factors are marked in bold (**p < 0.05**). nTL = the parents’ TL when they were nestlings (i.e., 10–13 days after hatching). See below for full model.

| ***Fixed effects*** | *F* | *df* | | *p* | | *Eta2 (η^2^) (partial)* | |
| --- | --- | --- | --- | --- | --- | --- | --- |
| Parental pair group | 3.391 | 1, 29.826 | | 0.076 | | 0.10 | |
| Parental age group | 0.080 | 1, 29.295 | | 0.779 | | < 0.01 | |
| Body mass at day10 | 0.070 | 1, 54.505 | | 0.792 | | < 0.01 | |
| ***Random effects*** | *Variance* | | *Std.Dev* | | *# group* | | *# observations* |
| Cage | 0.034 | | 0.183 | | 33 | | 59 |
| Residuals | 0.053 | | 0.23 | |  | |  |
|  |  | |  | |  | |  |

**Full model**. Same as above but including all two-way and three-way interactions.

| ***Fixed effects*** | *F* | *df* | | *p* | | *Eta2 (η^2^) (partial)* | |
| --- | --- | --- | --- | --- | --- | --- | --- |
| Parental pair group | 3.238 | 1, 29.218 | | 0.082 | | 0.10 | |
| Parental age group | 0.119 | 1, 28.776 | | 0.732 | | < 0.01 | |
| Body mass at day10 | 0.071 | 1, 53.444 | | 0.791 | | < 0.01 | |
| Parental pair group × Parental age group | 0.334 | 1, 28.708 | | 0.568 | | 0.01 | |
| ***Random effects*** | *Variance* | | *Std.Dev* | | *# group* | | *# observations* |
| Cage | 0.036 | | 0.190 | | 33 | | 59 |
| Residuals | 0.052 | | 0.229 | |  | |  |
|  |  | |  | |  | |  |

**Table S8.** Results from a Kruskal-Wallis test investigating if **blood sampling age of nestlings** (range 10–13 days after hatching) differed between parental pair group (long or short nTL), parental age group (young or old) or offspring sex. Significant factors are marked in bold (**p < 0.05**). nTL = the parents’ TL when they were nestlings.

| ***Dependent variables*** | ***Factor*** | | *Chi-sq* | | *df* | *p* | | |
| --- | --- | --- | --- | --- | --- | --- | --- | --- |
| *Sampling age* | | |  | |  |  | | |
| Parental pair group | | 1.272 | | 1 | | | 0.259 |  |
| Parental age group | | 2.236 | | 1 | | | 0.135 |  |
| Offspring sex | | 0.144 | | 1 | | | 0.705 |  |
|  | | |  | |  |  | | |
|  | | |  | |  |  | | |

**Table S9.** Determinants of nestling body mass on day10 after hatching. Results from a linear mixed-effects model (LMM) testing whether **nestling body mass** was affected by parental pair group (long or short nTL), parental age group (young or old) or nestling sex. Significant fixed effects are marked in bold (**p < 0.05**). nTL = the parents’ TL when they were nestlings.

| ***Fixed effects*** | | *F* | | *df* | | | *p* | | *Eta2 (η^2^) (partial)* | |  |
| --- | --- | --- | --- | --- | --- | --- | --- | --- | --- | --- | --- |
| Parental pair group | | 0.030 | | 1, 32.839 | | | 0.864 | | < 0.01 | |  |
| Parental age group | | 0.166 | | 1, 32.195 | | | 0.686 | | < 0.01 | |  |
| Nestling sex | | 1.857 | | 1, 87.487 | | | 0.176 | | 0.02 | |  |
| ***Random effects*** | | *Variance* | | | *Std.Dev* | | *# group* | | | *# observations* | |
| Cage | | 0.785 | | | 0.886 | | 41 | | | 108 | |
| Sampling age | | 4.202 | | | 2.050 | | 4 | | |  | |
| Residuals | | 1.519 | | | 1.232 | |  | | |  | |
|  | |  | | |  | |  | |  | |  |

***Table S10.*** Results from Kruskal-Wallis tests investigating if ***hatching success*** or ***mortality of nestlings*** differed depending on parental pair group (long or short nTL) or parental age group (young or old). Significant factors are marked in bold (**p < 0.05**). nTL = the parents’ TL when they were nestlings.

| ***Dependent variables*** | ***Factor*** | *Chi-sq* | *df* | *p* |
| --- | --- | --- | --- | --- |
| *Hatching success:* | |  |  |  |
| Parental nTL group | | 0.511 | 1 | 0.475 |
| Parental age group | | 0.204 | 1 | 0.652 |
|  | |  |  |  |
| *Mortality rate:* | |  |  |  |
| Parental nTL group | | 0.698 | 1 | 0.404 |
| Parental age group | | 1.067 | 1 | 0.302 |

***Table S11****.* Results from Kruskal-Wallis tests investigating if ***clutch size*** or ***number of infertile eggs*** (showing no embryo development at day 6) differed between clutch number, parental pair group (long or short nTL) or parental age group (young or old). Clutch number differed with respect to the incubation of the eggs: embryos from the 1st clutch were incubated artificially, while nestlings from the 2nd clutch were incubated naturally by parent birds. Significant factors are marked in bold (**p < 0.05)**. nTL = the parents’ TL when they were nestlings.

| ***Dependent variables*** | ***Factor*** | *Chi-sq* | *df* | *p* |
| --- | --- | --- | --- | --- |
| *Clutch size:* | |  |  |  |
| Development stage | | 1.343 | 1 | 0.247 |
| Parental pair group | | 0.480 | 1 | 0.489 |
| Parental age group | | 0.598 | 1 | 0.440 |
|  | |  |  |  |
| *Infertility:* | |  |  |  |
| Development stage | | 20.589 | 1 | **<0.001** |
| Parental pair group | | 0.291 | 1 | 0.590 |
| Parental age group | | 0.141 | 1 | 0.707 |

A greater number of eggs in the first breeding round (when embryo tissue was collected) was not fertile (approximately 38.7% of the eggs of a clutch) compared to the second breeding round (when nestlings were measured) (approximately 7.1%). This is likely due to inexperience in breeding (see Discussion).

**Linear mixed model (LMM) analyses using parental age and parental telomere length as continuous variables**

We repeated the analyses described in the Methods using parental TL and parental age as continuous variables (instead of dividing them into categorical variables (short/long and young/old)). We used offspring telomere length (TL; log-transformed t/s-ratio) as dependent variable and mid-parent nTL (average of log-transformed TL of mother and father at nestling stage), developmental stage, mid-parent age (average age of mother and father in days), offspring sex and their interactions as predictors. As in the other models, ‘cage’ was included as a random factor to control for shared parents and rearing environment. The significant interaction term between developmental stage and mid-parent nTL (F_1,205.03_ = 8.03, p = 0.005) (Table S12) confirmed that the relationship between parental nTL and offspring TL depends on the developmental stage (embryo or nestling). Separate LMMs for embryos and nestlings showed that, for embryos there was no association between mid-parent nTL and embryo TL (F_1,36.8_=0.26, p=0.62, Table S13). For nestlings there was a significant interaction between the two predictors mid-parental nTL and offspring sex (F_1,89.69_=4.0, p=0.048, Table S14). The latter corroborates that nestling sons’ and daughters’ TL were differently affected by our assortative mating design. In sum, analyses based on continuous parental nTL and parental age variables showed patterns that were consistent with the models using categorical parental nTL (i.e., parental pair group) and parental age group variables as predictors (Tables S4 to S7).

***Table S12.*** Determinants of ***offspring telomere length (TL)*** based on ***mid-parent measures***. **Reduced model** showing the results of a linear mixed model (LMM) testing the dependence of offspring telomere length (TL; log-transformed t/s-ratio) on mid-parent nTL (average of log-transformed TL of mother and father at nestling stage), mid-parent age (average age of mother and father in days), development stage (embryo or nestling), and offspring sex (son or daughter), as well as the interactions where p < 0.10. Significant factors are marked in bold (**p < 0.05**). nTL = the parent’ TL when they were nestlings (i.e., 10–13 days after hatching).

| ***Fixed effects*** | *F* | *Df* | *P* | *Eta2 (η) (partial)* |
| --- | --- | --- | --- | --- |
| **Mid-parent nTL** | **15.553** | **1, 37.464** | **< 0.001 ***** | **0.29** |
| Mid-parent age | 0.111 | 1, 37.806 | 0.741 | < 0.01 |
| **Development stage** | **4.624** | **1, 202.762** | **0.033 *** | **0.02** |
| Offspring sex | 2.809 | 1, 202.183 | 0.095 | 0.01 |
| **Mid-parent nTL × Development stage** | **8.031** | **1, 205.034** | **0.005 **** | **0.04** |
| Mid-parent nTL × Offspring sex | 2.973 | 1, 208.312 | 0.086 | 0.01 |
| Residuals |  |  |  |  |
| ***Random effects*** | *Variance* | *Std.Dev* | *# group* | *# observations* |
| Cage | 0.006 | 0.077 | 41 | 218 |
| Residuals | 0.056 | 0.236 |  |  |

***Table S13.*** Determinants of ***embryo telomere length (TL)*** based on ***mid-parent measures***. **Reduced model** showing the results of a linear mixed model (LMM) testing the dependence of embryo telomere length (TL; log-transformed t/s-ratio) on mid-parent nTL (average of log-transformed TL of mother and father at nestling stage), mid-parent age (average age of mother and father in days), and offspring sex (son or daughter), as well as the interactions where p < 0.10. Significant factors are marked in bold (**p < 0.05**). nTL = the parent’ TL when they were nestlings (i.e., 10 – 13 days after hatching).

| ***Fixed effects*** | *F* | *Df* | *p* | *Eta2 (η) (partial)* |
| --- | --- | --- | --- | --- |
| Mid-parent nTL | 0.255 | 1, 36.796 | 0.617 | < 0.01 |
| Mid-parent age | 0.780 | 1, 35.977 | 0.383 | 0.02 |
| Offspring sex | 1.181 | 1, 96.973 | 0.280 | 0.01 |
|  |  |  |  |  |
| ***Random effects*** | *Variance* | *Std.Dev* | *# group* | *# observations* |
| Cage | 0.017 | 0.131 | 36 | 110 |
| Residuals | 0.036 | 0.189 |  |  |

***Table S14.*** Determinants of ***nestling telomere length (TL)*** based on ***mid-parent measures***. **Reduced model** showing the results of a linear mixed model (LMM) testing the dependence of nestling telomere length (TL; log-transformed t/s-ratio) on mid-parent nTL (average of log-transformed TL of mother and father at nestling stage), mid-parent age (average age of mother and father in days), and offspring sex (son or daughter), as well as the interactions where p < 0.10. Significant factors are marked in bold (**p < 0.05**). nTL = the parent’ TL when they were nestlings (i.e., 10–13 days after hatching).

| ***Fixed effects*** | *F* | *Df* | *p* | *Eta2 (η) (partial)* |
| --- | --- | --- | --- | --- |
| **Mid-parent nTL** | **16.677** | **1, 34.584** | **< 0.001***** | **0.33** |
| Mid-parent age | 0.959 | 1, 332.153 | 0.335 | 0.03 |
| Offspring sex | 1.748 | 1, 82.596 | 0.190 | 0.02 |
| Body mass at day10 | 0.357 | 1, 101.693 | 0.551 | < 0.01 |
| **Mid-parent nTL × Offspring sex** | **4.006** | **1, 89.698** | **0.048*** | **0.04** |
|  |  |  |  |  |
| ***Random effects*** | *Variance* | *Std.Dev* | *# group* | *# observations* |
| Cage | 0.020 | 0.141 | 41 | 108 |
| Residuals | 0.052 | 0.227 |  |  |


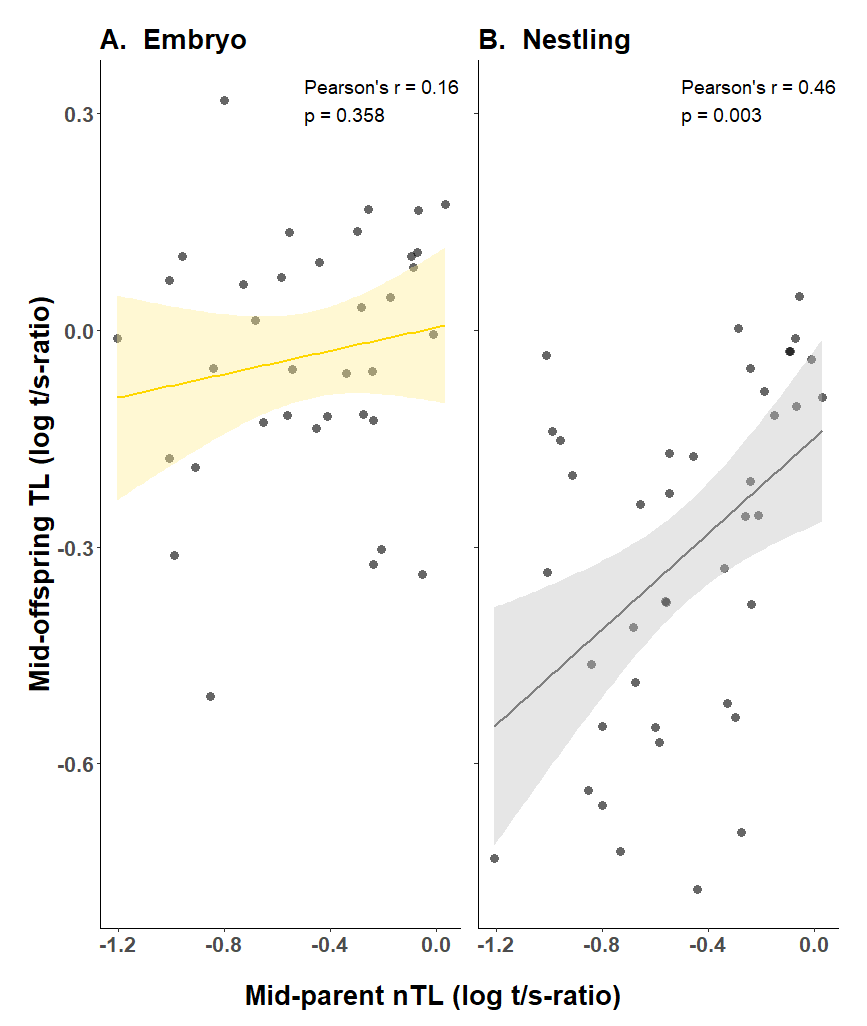


***Figure S1.*** Separate mid-parent – mid-offspring regression of log-transformed telomere length (TL; measured as t/s-ratio) for offspring at embryo and nestling stages for visualisation. The regression estimates the relationship between the mean parent nTL and mean offspring TL at either the embryo or the nestling stage. The solid line represents the linear trend, with 95% confidence intervals shaded. nTL = the parents’ TL when they were nestlings.


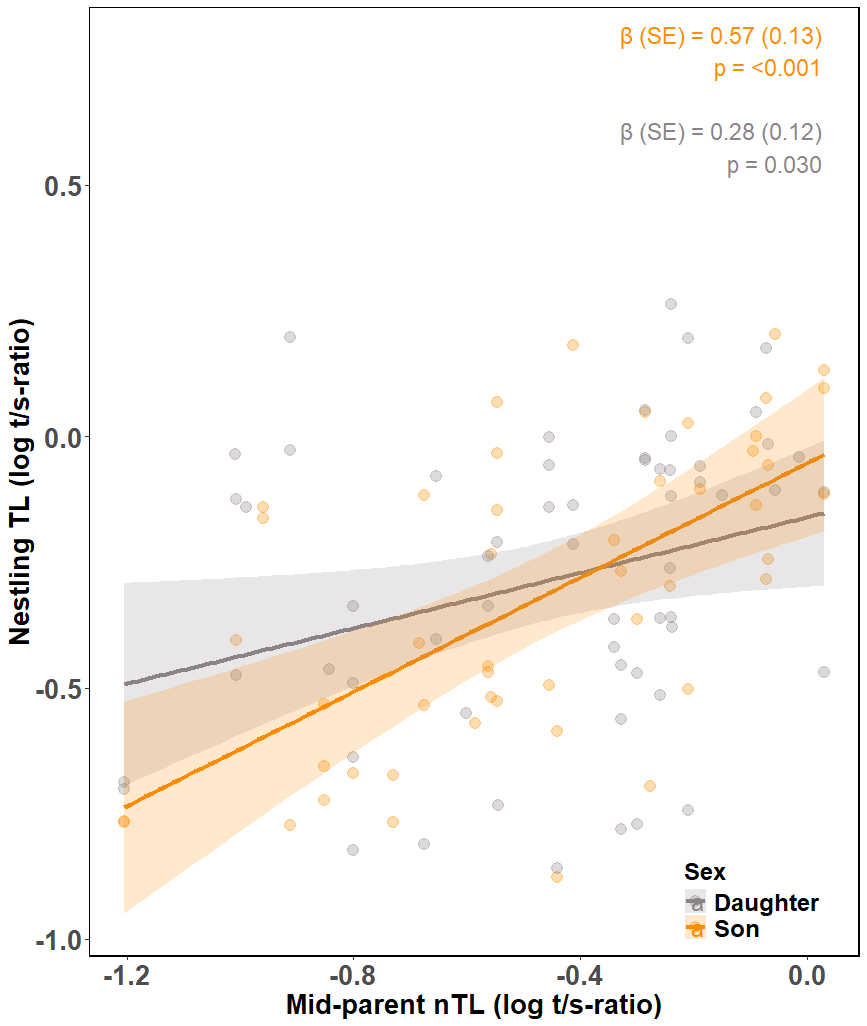


***Figure S2.*** Relationship of mid-parent nTL (average of log-transformed TL of mother and father at nestling stage) and nestling TL (log-transformed t/s-ratio) for sons (orange) and daughters (grey). Points show raw data, lines indicate slopes predicted from the LMM, and shaded ribbons represent 95% confidence intervals. Estimated slopes (β), SE and corresponding p-values are shown within the panels for each sex, adjusted for parental age and nestling mass.

**
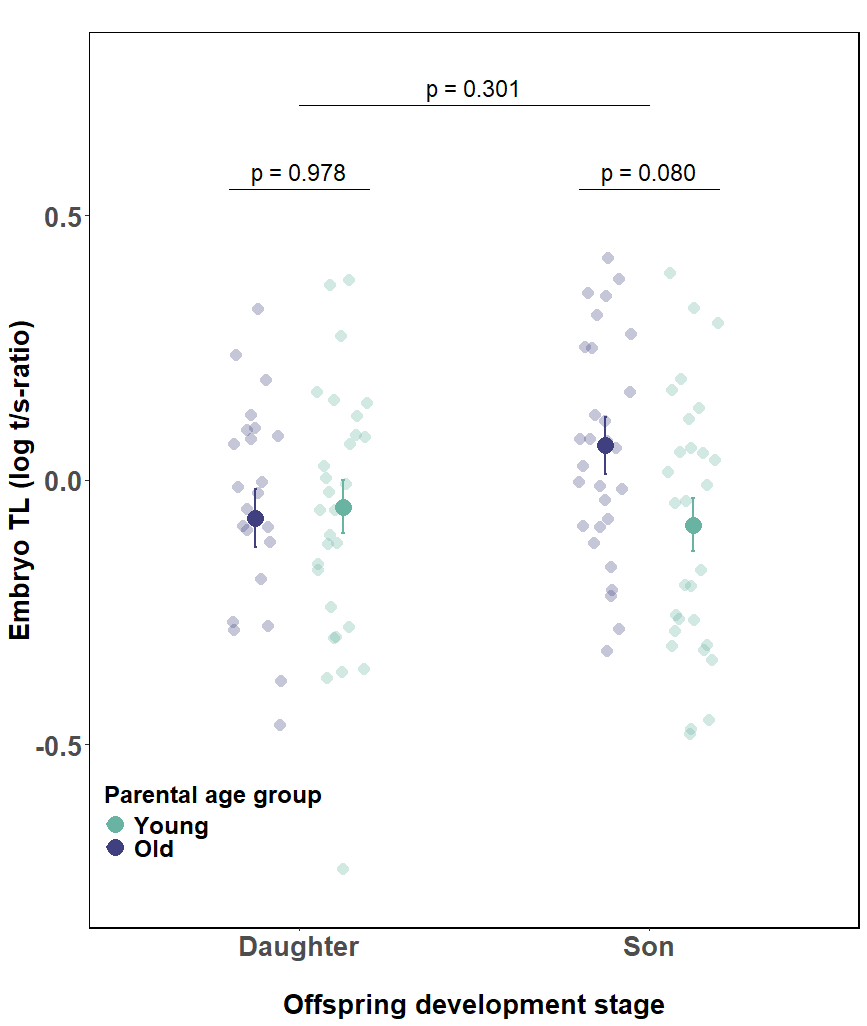
**

**Figure S3.** Embryo telomere length for sons and daughters from parents with **old** (purple) and **young** (green) age groups. Large points and error bars represent means ± S.E. of log-transformed t/s-ratio values, while semi-transparent small points show raw data. Significance differences between offspring sex group are determined using *post hoc* least squares means pairwise comparisons.
